# A Revised Bimodal Generalized Extreme Value Distribution: Theory and Climate Data Application

**DOI:** 10.3390/e27070749

**Published:** 2025-07-14

**Authors:** Cira E. G. Otiniano, Mathews N. S. Lisboa, Terezinha K. A. Ribeiro

**Affiliations:** Statistics Department, University of Brasília, Brasília 70910-900, DF, Brazilterezinha.ribeiro@unb.br (T.K.A.R.)

**Keywords:** heterogeneous data, bimodal GEV distribution, properties

## Abstract

The bimodal generalized extreme value (BGEV) distribution was first introduced in 2023. This distribution offers greater flexibility than the generalized extreme value (GEV) distribution for modeling extreme and heterogeneous (bimodal) events. However, applying this model requires a data-centering technique, as it lacks a location parameter. In this work, we investigate the properties of the BGEV distribution as redefined in 2024, which incorporates a location parameter, thereby enhancing its flexibility in practical applications. We derive explicit expressions for the probability density, the hazard rate, and the quantile function. Furthermore, we establish the identifiability property of this new class of BGEV distributions and compute expressions for the moments, the moment-generating function, and entropy. The applicability of the new model is illustrated using climate data.

## 1. Introduction

The Fréchet, Weibull, and Gumbel extreme value distributions [1,2] are genuine probabilistic models for extreme event data, as they correspond to the asymptotic distribution of statistics extreme of independent and identically distributed random variables. The generalized extreme value (GEV) distribution, presented by [3], summarizes the three extreme distributions. For this reason, the GEV distribution is widely used to model extreme events across various fields, including insurance, finance, and hydrology. The theory and applications of the GEV distribution are thoroughly discussed in the books [4,5,6,7,8,9], among others.

A continuous random variable *X* has a GEV distribution, *X*∼F(.;ξ,μ,σ), if its cumulative distribution function (CDF) and probability density function (PDF) are given, respectively, by 

(1) $$\begin{array}{c} F\left(y;\xi ,\mu ,\sigma \right)=\left\{\begin{array}{cc} \exp\left\{-{\left[1+\xi \left(\frac{y-\mu }{\sigma }\right)\right]}^{-1/\xi }\right\}, & \;\;\;\mathrm{if}\;\xi \neq 0,\\ \exp\left\{-\exp\left[-\left(\frac{y-\mu }{\sigma }\right)\right]\right\}, & \;\;\;\mathrm{if}\;\xi =0, \end{array}\right. \end{array}$$

and

(2) $$f\left(y;\xi ,\mu ,\sigma \right)=\left\{\begin{array}{cc} \frac{1}{\sigma }{\left[1+\xi \left(\frac{y-\mu }{\sigma }\right)\right]}^{-1/\xi -1}\exp\left\{-{\left[1+\xi \left(\frac{y-\mu }{\sigma }\right)\right]}^{-1/\xi }\right\}, & \;\;\;\mathrm{if}\;\xi \neq 0,\\ \frac{1}{\sigma }\exp\left\{-\left(\frac{y-\mu }{\sigma }\right)-\exp\left[-\left(\frac{y-\mu }{\sigma }\right)\right]\right\}, & \;\;\;\mathrm{if}\;\xi =0, \end{array}\right.$$

with shape parameter ξ∈R, scale parameter σ>0, and location parameter μ∈R.

The support of the GEV distribution depends on the values of the parameters. It is the set $\left\{x\in R:[1+\xi \left(\frac{y-\mu }{\sigma }\right)]\right\}$ for ξ≠0 and R for ξ=0.

The parameter ξ determines the weight of the tail of the distribution. The GEV distribution accommodates heavy-tailed and light-tailed distributions and is characterized by its unimodal shape. Some of the unimodal generalizations of the GEV distribution are the transmuted GEV distribution [10,11], the dual gamma generalized extreme value distribution, the exponentiated generalized extreme value distribution [11], and the blended generalized extreme value distribution [12].

In various applications, extreme climate data, such as wind speed, humidity, and temperature, exhibit heterogeneous (bimodal) densities with rare events and heavy tails. A very promising model for extreme heterogeneous data is the bimodal GEV distribution, as defined in [13].

Following [13], a  random variable *X* has a bimodal GEV (BGEV) distribution; $X∼F_{\mathrm{BGEV}}\left(\cdot ;\xi ,\mu ,\sigma ,\delta \right)$ if its cumulative distribution function is given by

(3) $$\begin{array}{cc} F_{\mathrm{BGEV}}(x; & \xi ,\mu ,\sigma ,\delta )=F_{\xi ,\mu ,1}\left(T_{\sigma ,\delta }\left(x\right)\right), \end{array}$$

with ξ∈R, μ∈R, σ>0, δ>−1, and the transformation $T_{\sigma ,\delta }\left(\cdot \right)$ is defined by

(4) $$\begin{array}{c} T_{\sigma ,\delta }\left(x\right)=\sigma x{\left|x\right|}^{\delta }\;,\;x\in R, \end{array}$$

is invertible and differentiable. The support of the probability density function associated with the distribution (3) depends on the shape parameter. When ξ=0, the support is R, when ξ>0, the interval is $\left[\mathrm{sgn}\left(\mu -\frac{1}{\xi }\right){\left(|\mu -\frac{1}{\xi }|\sigma^{-1}\right)}^{\frac{1}{\delta +1}},+\infty \right)$, and when ξ<0, the interval is $\left(-\infty ,\mathrm{sgn}\left(\mu -\frac{1}{\xi }\right){\left(|\mu -\frac{1}{\xi }|\sigma^{-1}\right)}^{\frac{1}{\delta +1}}\right]$, where $\mathrm{sgn}\left(x\right)=\frac{\left|x\right|}{x}$.

The disadvantage of the model (3) is that its four parameters are shape parameters. In this distribution, there are no location and scale parameters. In other words, σ is not a scale parameter and μ is not a location parameter, as is the case with the GEV distribution. Furthermore, the local minimum of the PDF is always located at zero. This limitation complicates its applicability, as real bimodal data can have local minimum at any value of the real line.

The chief goal of this paper is to examine several properties of the new BGEV distribution, which was redefined by [14] and which includes a location parameter, and to illustrate its applicability. Specifically, this paper complements the work of [14] in three directions. First, it presents the proof of the identifiability of the new bimodal GEV, which is crucial for the practical application of this model. Second, it presents expressions for the moments, the moment-generating function, and the differential entropy of the new BGEV model. Third, it presents a real data application of the BGEV distribution in a scenario where a bimodal model for extreme data is needed.

The remainder of this paper is structured as follows. Section 2 presents the main results of this work. We begin in Section 2.1 with the definition of the main functions related to the new BGEV model. Next, in Section 2.2, we provide a graphical illustration of the new BGEV model. Finally, in Section 2.3, we show the main properties of the new BGEV distribution. Section 3 contains an application of the new bimodal BGEV model to climate data. Finally, Section 4 closes the paper with some concluding remarks.

## 2. The New BGEV Distribution

Initially, in Section 2.1, we show how the model (3) was redefined by [14], presenting the cumulative distribution function, probability density function, failure rate function, and quantile function. The versatility of the BGEV distribution is illustrated through the graphical representations in Section 2.2. The main results of this work are the properties of the new BGEV distribution, which are in Section 2.3.

### 2.1. The Redefined BGEV Distribution

**Definition 1.**  *A random variable X has a bimodal GEV distribution with location parameter X∼BGEV(ξ,μ,σ,δ) if  CDF is given by*

(5) F(x;ξ,μ,σ,δ)=F(T(x;μ,δ);ξ,0,σ),

*where*

(6) $$T\left(x;\mu ,\delta \right)=\left(x-\mu \right){\left|x-\mu \right|}^{\delta },\;\;\delta >-1,\mu \in R.$$

The inverse and derivative functions of T(·) are, respectively, given by

(7) $$T^{-1}\left(x;\mu ,\delta \right)=\mathrm{sgn}\left(x\right){\left|x\right|}^{1/(\delta +1)}+\mu$$

and

(8) $$\begin{array}{c} T^{\prime }\left(x;\mu ,\delta \right)=\left(\delta +1\right){\left|x-\mu \right|}^{\delta }. \end{array}$$

For simplicity, we write T(x;μ,δ)=T(x), where the notation with parameters is used only to show the role of the parameters σ and μ.

The expressions (7) and (8) allow us to obtain the following PDF of X∼BGEV(ξ,μ,σ,δ), given by

(9) $$\begin{array}{c} f\left(x;\xi ,\mu ,\sigma ,\delta \right)=\left\{\begin{array}{cc} \frac{1}{\sigma }{\left[1+\xi \left(\frac{T(x)}{\sigma }\right)\right]}^{(-1/\xi )-1}\exp\left[-{\left[1+\xi \left(\frac{T(x)}{\sigma }\right)\right]}^{-1/\xi }\right]T^{\prime }\left(x\right), & \;\;\;\xi \neq 0,\\ \frac{1}{\sigma }\exp\left(-\frac{T(x)}{\sigma }\right)\exp\left[-\exp\left(-\frac{T(x)}{\sigma }\right)\right]T^{\prime }\left(x\right), & \;\;\;\xi =0, \end{array}\right. \end{array}$$

whose support is

(10) $$\begin{array}{c} \mathrm{Support}\left(f\left(\cdot ;\xi ,\mu ,\sigma ,\delta \right)\right)=\left\{\begin{array}{cc} \left[\mu -{\left(\frac{\sigma }{\xi }\right)}^{1/\left(\delta +1\right)},+\infty \right), & \mathrm{if}\;\xi >0,\\ \left(-\infty ,\mu +{\left|\frac{\sigma }{\xi }\right|}^{1/\left(\delta +1\right)}\right], & \mathrm{if}\;\xi <0,\\ (-\infty ,+\infty ), & \mathrm{if}\;\xi =0. \end{array}\right. \end{array}$$

In the BGEV model, ξ, δ, and σ are shape parameters, while μ is a location parameter. It is important to note that in the GEV distribution (1), σ is a scale parameter; however, in (5), σ is not a scale parameter, because does not satisfy the condition

$$f\left(x;\xi ,\mu ,\sigma ,\delta \right)=\frac{1}{\sigma }f\left(\frac{x}{\sigma };\xi ,\mu ,1,\delta \right),$$

since $\frac{1}{\sigma }T\left(x;\mu ,\delta \right)\neq T\left(\frac{x}{\sigma };\mu ,\delta \right)$.

On the other hand, the parameter μ in (5) is a location parameter. To prove this, it suffices to observe that T(x;μ,δ)=T(x−μ;0,δ) and

$$\begin{array}{ccc} F(x;\xi ,\mu ,\sigma ,\delta ) & = & F(T(x-\mu ;0,\delta );\xi ,0,\sigma )\\ & = & F(T(x;\mu ,\delta );\xi ,\mu ,\sigma ). \end{array}$$

Thus, f(x;ξ,μ,σ,δ)=f(x−μ;ξ,0,σ,δ).

The model (5) is a generalization of the GEV distribution, because when δ=0 the BGEV distribution returns to GEV distribution. That is, X∼BGEV(ξ,μ,σ,0)=GEV(ξ,μ,σ).

From the expressions in (5) and (9), it is simple to obtain the survival and hazard functions. These functions are useful in the area of reliability and for calculating risk measures in other areas. The survival and hazard functions are given by the expressions

$$\begin{array}{ccc} S(y) & = & \left\{\begin{array}{cc} 1-\exp\left[-{\left[1+\xi \left(\frac{T(y)}{\sigma }\right)\right]}^{-1/\xi }\right], & \xi \neq \;0\\ 1-\exp\left[-\exp\left[-\frac{T(y)}{\sigma }\right]\right], & \xi =0 \end{array}\right. \end{array}$$

and

$$\begin{array}{ccc} h(y) & = & \left\{\begin{array}{cc} \frac{\frac{1}{\sigma }{\left[1+\xi \left(\frac{T(y)}{\sigma }\right)\right]}^{(-1/\xi )-1}\exp\left[-{\left[1+\xi \left(\frac{T(y)}{\sigma }\right)\right]}^{-1/\xi }\right]T^{\prime }\left(y\right)}{1-\exp\left[-{\left[1+\xi \left(\frac{T(y)}{\sigma }\right)\right]}^{-1/\xi }\right]}, & \xi \neq \;0\\ \frac{\exp\left(-\frac{T(y)}{\sigma }\right)\exp\left[-\exp\left(-\frac{T(y)}{\sigma }\right)\right]T^{\prime }\left(y\right)}{1-\exp\left[-\exp\left[-\frac{T(y)}{\sigma }\right]\right]}, & \xi =0, \end{array}\right. \end{array}$$

respectively. The support of the survival and hazard functions is the set (10).

An important property of the new BGEV model is that its quantile function has a simple closed-form expression. This feature is extremely useful for simulation procedures and the calculation of risk measures in various applied fields.

From (1), (5), and (7), we have that the quantile function of BGEV model given by

$$\begin{array}{ccc} Q(y) & = & \left\{\begin{array}{cc} \mathrm{sgn}\left\{\frac{\sigma }{\xi }\left[{\left(-\log\left(y\right)\right)}^{-\xi }-1\right]\right\}{\left|\frac{\sigma }{\xi }\left[{\left(-\log\left(y\right)\right)}^{-\xi }-1\right]\right|}^{1/\left(\delta +1\right)}+\mu , & \mathrm{if}\;\;\xi \neq 0\\ \mathrm{sgn}\left[-\sigma \log\left(-\log\left(y\right)\right)\right]\;{\left|-\sigma \log\left(-\log\left(y\right)\right)\right|}^{1/\left(\delta +1\right)}+\mu , & \mathrm{if}\;\;\xi =0. \end{array}\right. \end{array}$$

### 2.2. Graphic Illustrations of New BGEV Distribution

**Figure 1:**
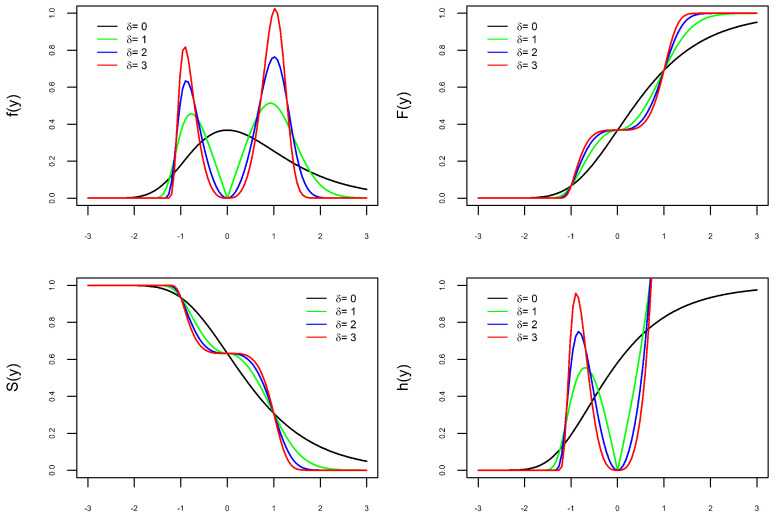
Graphs of X∼BGEV(0,0,1,δ) with δ varying: PDF (**top left**), CDF (**top right**), Survival (**bottom left**), and Hazard (**bottom right**).

**Figure 2:**
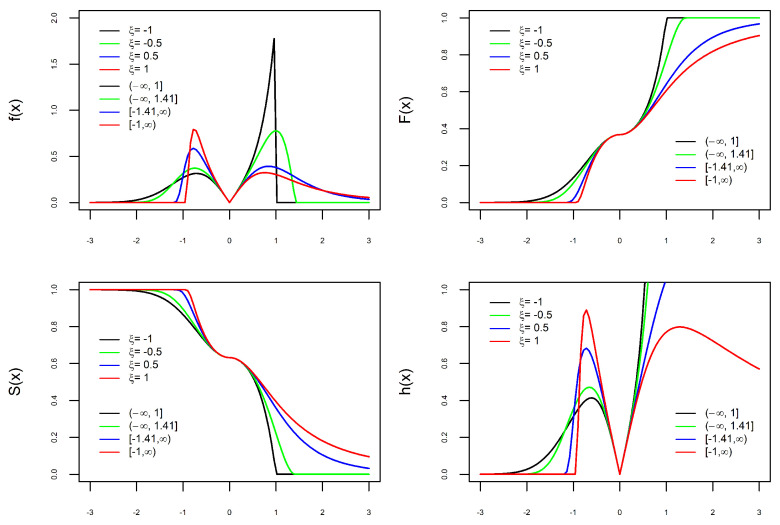
Graphs of X∼BGEV(ξ,0,1,1) with ξ varying: PDF (**top left**), CDF (**top right**), Survival (**bottom left**), and Hazard (**bottom right**).

**Figure 3:**
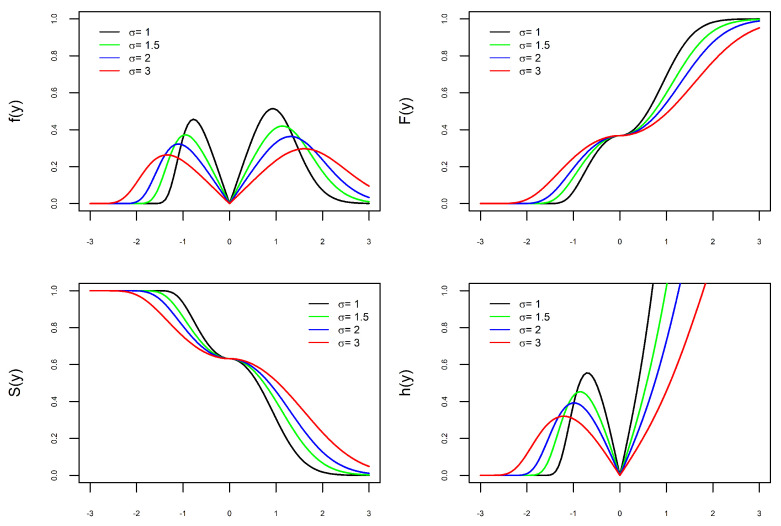
Graphs of X∼BGEV(0,0,σ,1) with σ varying. PDF (**top left**), CDF (**top right**), Survival (**bottom left**), and Hazard (**bottom right**).

**Figure 4:**
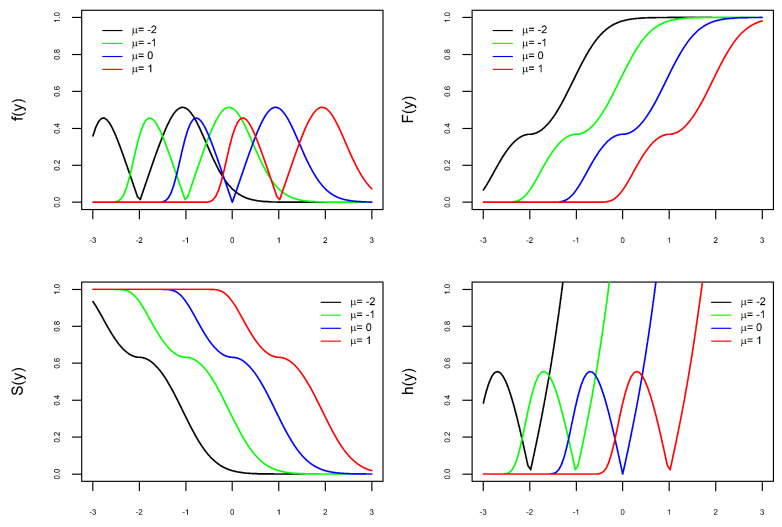
Graphs of X∼BGEV(0,μ,1,1) with μ varying: PDF (**top left**), CDF (**top right**), Survival (**bottom left**), and Hazard (**bottom right**).

The versatility of the new PDF, defined in (9), is illustrated in Figure 1, Figure 2, Figure 3 and Figure 4. Depending on the combination of parameters, the PDF can be unimodal or bimodal, symmetric or asymmetric, and have a heavy or light tail. To better understand the role of each of the four parameters in the PDF, we consider four scenarios. In each scenario, we fix three parameters and let the fourth parameter vary to understand its effect on the curves. In each of the Figure 1, Figure 2, Figure 3 and Figure 4 are the graphs of the PDF (f), CDF (F), survival (S), and hazard functions (h) of X∼BGEV(ξ,μ,σ,δ).

In Figure 1, Figure 2 and Figure 3, the graphs of the four functions change as the values of ξ, δ, and σ vary. This illustrates our comment above that the parameters ξ, δ, and σ are shape parameters. Figure 1 shows the effect of the parameter δ on the curves. When δ=0, the PDF is unimodal, whereas it is bimodal for δ>0. Furthermore, the larger the value of δ, the further apart and larger the modes are and the heavier the tails. The effect of the parameter ξ on the curves is shown in Figure 2. As ξ increases, the density tails are heavier and the asymmetry becomes more evident. In Figure 3, one can see that the parameter σ also modifies the PDF. The parameter σ is not a scale parameter, since the PDF remains fixed at the local minimum. This confirms our proof above that σ is not a scale parameter. In Figure 4, the PDF only moves with the variation in μ. This also confirms that μ is a location parameter. Regarding the hazard function h, by depending on the combination of model parameters, the h function is increasing, decreasing, unimodal, N-shaped, or M-shaped. In other words, the BGEV distribution is quite flexible for modeling data regarding survival/reliability.

As previously demonstrated, the mode of distribution is governed by the parameter δ>0. In practical applications, extreme data from heterogeneous populations can be appropriately modeled using the BGEV distribution with δ>0, reflecting its ability to capture bimodal patterns. For instance,  [13] fitted this distribution to maximum wind speed and maximum temperature data, obtaining δ values of 0.9434 and 0.4201, respectively, which clearly indicate the presence of bimodality in the extremes. This characteristic is consistent with the seasonal behavior of the studied region, which exhibits two well-defined climatic seasons (wet and dry), each associated with distinct regimes of extreme event occurrences, resulting in two distinct modes throughout the year.

### 2.3. Properties

In statistics, identifiability is an important property that a family of distributions must satisfy for accurate inference. A distribution of a family is identifiable if different values of the parameters produce different probability distributions. In other words, the parameter of the distribution is unique. The following shows that the family of distributions of F(·;ξ,0,σ,δ) in (5) is identifiable. In addition, we derive other properties, including formulas for the moments and quantile functions.

#### 2.3.1. Identifiability

Let F={F=F(·;θ)} be a family of CDFs. This class F is identifiable if and only if for any $F_{1}=F\left(\cdot ;\theta_{1}\right),F_{2}=F\left(\cdot ;\theta_{2}\right)\in F$, the equality $F_{1}=F_{2}$ implies $\theta_{1}=\theta_{2}$.

**Proposition 1.**  *The family of BGEV distributions with known parameter δ; $F_{BGEV}=\left\{F\left(x;\xi ,0,\sigma ,\delta \right):F\left(x;\xi ,0,\sigma ,\delta \right)\;as\;\left(5\right)\right\}$ is identifiable.*

**Proof.**  The authors of [15] demonstrated the identifiability of the finite mixture of GEV distributions, particularly that the family of a GEV component is identifiable. That is, the family $F_{G}=\left\{F\left(\cdot ;\xi ,\mu ,\sigma \right):F\left(\cdot ;\xi ,\mu ,\sigma \right)\;\mathrm{as}\;\left(1\right)\right\}$ is identifiable. Thus, to prove that for any $F\left(\cdot ;\xi_{1},0,\sigma_{1},\delta \right)),\;F\left(\cdot ;\xi_{2},0,\sigma_{2},\delta \right)\in F_{BGEV}$ equality

(11) $$\begin{array}{c} F\left(x;\xi_{1},0,\sigma_{1},\delta \right)=F\left(x;\xi_{2},0,\sigma_{2},\delta \right),\;\;\forall x\in \mathrm{Support}(F), \end{array}$$

implies $\xi_{1}=\xi_{2},\sigma_{1}=\sigma_{2}$. From (5), we have that (11) is equivalent to

(12) $$\begin{array}{c} {F(x|x|}^{\delta };\xi_{1},0,\sigma_{1}{)=F(x|x|}^{\delta };\xi_{2},0,\sigma_{2}). \end{array}$$

Since the function $F_{G}$ is identifiable, it follows from (12) that the equality necessarily implies $\xi_{1}=\xi_{2}$ and $\sigma_{1}=\sigma_{2}$.    □

#### 2.3.2. Moments and Moment Generating Function

To calculate the moments of X∼BGEV(ξ,μ,σ,δ), first consider the gamma function, the upper incomplete gamma function, and the lower incomplete gamma function, defined according to [16], respectively, by

(13) $$\begin{array}{cc} \Gamma (a) & :=\int_{0}^{\infty }t^{a-1}e^{-t}dt \end{array}$$

(14) $$\begin{array}{cc} \Gamma \left(a,x\right) & :=\int_{x}^{\infty }t^{a-1}e^{-t}dt \end{array}$$

and

(15) $$\begin{array}{cc} \gamma \left(a,x\right) & :=\int_{0}^{x}t^{a-1}e^{-t}dt, \end{array}$$

where $a\in R^{+}$.

**Proposition 2.**  *Let X∼BGEV(ξ,μ,σ,δ) with ξ≠0, then the k-th integer moment of X is given by*

(16) E(Xk)=∑j=0kkj(−1)(k−j)(δ+2)δ+1σξk−jδ+1∑i=0[|k−jδ+1|][|k−jδ+1|]i(−1)iγ1−ξ[|k−jδ+1|]−i,1+∑j=0kkjσξk−jδ+1∑i=0[|k−jδ+1|][|k−jδ+1|]i(−1)iΓ1−ξ[|k−jδ+1|]−i,1,

*when ξ>0, whenever $\xi <\frac{\delta +1}{k}$ and*

E(Xk)=∑j=0kkj(−1)(k−j)(δ+2)δ+1σξk−jδ+1∑i=0[|k−jδ+1|][|k−jδ+1|]i(−1)iΓ1−ξ[|k−jδ+1|]−i,1+∑j=0kkjσξk−jδ+1∑i=0[|k−jδ+1|][|k−jδ+1|]i(−1)iγ1−ξ[|k−jδ+1|]−i,1,

*when ξ<0, whenever $\xi >-\frac{\delta +1}{k}$.*

**Proof.**  By definition

(17) $$\begin{array}{ccc} E(X^{k}) & = & \int_{-\infty }^{+\infty }x^{k}f\left(T\left(x\right);\xi ,0,\sigma \right)T^{\prime }\left(x\right)dx, \end{array}$$

where f(·;ξ,0,σ) is defined in (2), *T* as in (6), and $T^{\prime }$ given in (8). By substituting y=T(x) into (17), the moments are expressed as follows:

(18) $$\begin{array}{ccc} E(X^{k}) & = & \int_{-\infty }^{+\infty }{\left[\mathrm{sng}\left(y\right)|y\right|}^{\frac{1}{\delta +1}}{+\mu ]}^{k}f\left(y;\xi ,0,\sigma \right)dy. \end{array}$$

As $k\in Z^{+}$, the Newton Binomial formula is used, so (18) is updated by the integral

(19) E(Xk)=∑j=0kkjμj∫−∞+∞[sng(y)]k−j|y|k−jδ+1f(y;ξ,0,σ)dy=∑j=0kkjμj(−1)(k−j)(δ+2)δ+1EYk−jδ+1I[Y<0]+∑j=0kkjμjEYk−jδ+1I[Y≥0],

where Y∼GEV(ξ,0,σ) and $I_{A}$ is the indicator function of the set *A*; $I_{A}\left(\omega \right)=1$ if ω∈A and $I_{A}\left(\omega \right)=0$ otherwise. Now, we need to analyze the cases where ξ>0 and ξ<0.

Case ξ>0: By replacing (2) in (19) and changing the variable $t={\left[1+\frac{\xi }{\sigma }y\right]}^{-1/\xi }$ for $r=[|\frac{k-j}{\delta +1}|]$, it follows that

(20) $$\begin{array}{ccc} E(Y^{k}I_{\left[Y\geq 0\right]}) & = & \int_{0}^{+\infty }y^{r}\frac{1}{\sigma }{\left[1+\frac{\xi }{\sigma }y\right]}^{-\frac{1}{\xi }-1}\exp\left\{-{\left[1+\frac{\xi }{\sigma }y\right]}^{-\frac{1}{\xi }}\right\}dy\\ & = & \int_{1}^{+\infty }{\left(\frac{\sigma }{\xi }t^{-\xi }-\frac{\sigma }{\xi }\right)}^{r}e^{-t}dt. \end{array}$$

Newton’s Binomial is used in (20) and we obtain

(21) E(YkI[Y≥0])=∑i=0rri(−1)iσξr∫1+∞t−ξ(r−i)e−tdt.

In the same way, the *k*-th moment of *Y* truncated in the negative part is

(22) E(YkI[Y<0])=∫−σξ0yr1σ1+ξσy−1ξ−1exp−1+ξσy−1ξdy=∫01σξt−ξ−σξre−tdt=∑i=0rri(−1)iσξr∫01t−ξ(r−i)e−tdt.

The lower incomplete gamma function (15) and the upper incomplete gamma function (14) are used to represent the integrals of (21) and (22), respectively. Consequently, the proof of (16) follows by substituting these updates into Equation (19).

Case ξ<0: The same procedure as in the case where ξ>0 is repeated, respecting the support $\left\{y:y\in \left(-\infty -\frac{\sigma }{\xi }\right]\right\}$ of Y∼GEV(ξ,0,σ).    □

**Remark 1.**  *From Proposition 2, we have that for ξ>0, $E(X^{k})$ is finite for $k<\frac{\delta +1}{\xi }$. That is, the two shape parameters ξ and δ influence the weight of the tail of the new distribution. Consequently, the tail index of the new BGEV distribution is $\frac{\delta +1}{\xi }$. That is, the right tail of the BGEV distribution can be heavier than the tail of the GEV distribution.*

In the following corollary from Proposition 2, we obtain a known result.

**Corollary 1.**  *Let X∼BGEV(ξ,μ,σ,0)=GEV(ξ,μ,σ) where ξ≠0. The k-th moment of X is given by*

E(Xk)=∑j=0kkjμjσξk−jΓ(1−ξ(k−j)).

**Proof.**  From (19), when δ=0, we obtain

E(Xk)=∑j=0kkjμjEYk−j=E∑j=0kkjμjYk−j=E(Y+μ)k,

where Y∼GEV(ξ,0,σ) and Y+μ∼GEV(ξ,μ,σ). The proof ends with the use of the expressions (21) and (22) and the fact that Γ(x,s)+γ(x,s)=Γ(x).    □

The mean of a random variable X∼BGEV(ξ,μ,σ,δ) exists when ξ<δ+1. It is given in the following corollary.

**Corollary 2.**  *Let X∼BGEV(ξ,μ,σ,δ) with ξ≠0. Then, for ξ>0*

E(X)=(−1)δ+2δ+1σξ1δ+1∑i=011i(−1)iγ1−ξ1−i,1+σξ1δ+1∑i=011i(−1)iΓ1−ξ1−i,1,for ξ>0,

*and*

E(X)=(−1)δ+2δ+1σξ1δ+1∑i=011i(−1)iΓ1−ξ1−i,1+σξ1δ+1∑i=011i(−1)iγ1−ξ1−i,1,for ξ<0.

For ξ=0, an expression of the moment generating function was obtained. It is given in the following proposition.

**Proposition 3.**  *Let X∼BGEV(ξ,μ,σ,δ) with ξ=0. The moment generating function of X is given by*

(23) $$\begin{array}{c} M_{X}\left(t\right)=e^{\mu t}\sum_{k=0}^{\infty }\frac{t^{k}}{k!}\left[{\left(-1\right)}^{\frac{k(\delta +2)}{\delta +1}}E\left(Y^{\frac{k}{\delta +1}}I_{\left[Y<0\right]}\right)+E\left(Y^{\frac{k}{\delta +1}}I_{\left[Y\geq 0\right]}\right)\right], \end{array}$$

*where Y∼GEV(0,0,σ).*

**Proof.**  By definition, we have

(24) $$\begin{array}{c} M_{X}\left(t\right)=\int_{-\infty }^{+\infty }\frac{1}{\sigma }\exp\left\{tx\right\}\exp\left\{-\frac{T(x)}{\sigma }-\exp\left[-\frac{T(x)}{\sigma }\right]\right\}dx. \end{array}$$

When using the substitution y=T(x) in (24) and the fact that

$$x=\mathrm{sgn}\left(\ln y^{-\sigma }\right){\left|\ln y^{-\sigma }\right|}^{\frac{1}{\delta +1}}+\mu$$

we have that

(25) $$\begin{array}{c} M_{X}\left(t\right)=e^{\mu t}\int_{0}^{+\infty }\exp\left\{\mathrm{sgn}\left(\ln y^{-\sigma }\right){\left|\ln y^{-\sigma }\right|}^{\frac{1}{\delta +1}}t\right\}\exp\left\{-y\right\}dy. \end{array}$$

The new substitution $s=\ln(y^{-\sigma })$ allows you to update (25) by

(26) $$\begin{array}{c} M_{X}\left(t\right)=\frac{e^{\mu t}}{\sigma }\int_{-\infty }^{+\infty }\exp\left\{{\mathrm{sgn}\left(s\right)|s|}^{\frac{1}{\delta +1}}t\right\}\exp\left\{-\frac{s}{\sigma }-\exp\left[-\frac{s}{\sigma }\right]\right\}dy. \end{array}$$

Finally, the series representation of the exponential function is used. Thus, (26) is rewritten by the equation

$$\begin{array}{ccc} M_{X}\left(t\right) & = & e^{\mu t}\sum_{k=0}^{-\infty }\frac{t^{k}}{k!}{\left(-1\right)}^{\frac{k(\delta +2)}{\delta +1}}\int_{-\infty }^{0}\frac{1}{\sigma }s^{\frac{k}{\delta +1}}\exp\left\{-\frac{s}{\sigma }-\exp\left[-\frac{s}{\sigma }\right]\right\}ds\\ & + & e^{\mu t}\sum_{k=0}^{-\infty }\frac{t^{k}}{k!}\int_{0}^{+\infty }\frac{1}{\sigma }s^{\frac{k}{\delta +1}}\exp\left\{-\frac{s}{\sigma }-\exp\left[-\frac{s}{\sigma }\right]\right\}ds. \end{array}$$

   □

The following result is a particular case of (24). It coincides with the moment-generating function of the Gumbel distribution [6].

**Corollary 3.**  *Let X∼BGEV(ξ,μ,σ,0)=GEV(ξ,μ,σ). The moment generating function of X is given by*

(27) $$\begin{array}{c} M_{X}\left(t\right)=e^{\mu t}\Gamma \left(1-\sigma t\right). \end{array}$$

**Proof.**  When δ=0, the expression (23) reduces to

$$\begin{array}{ccc} M_{X}\left(t\right) & = & e^{\mu t}\sum_{k=0}^{\infty }\frac{t^{k}}{k!}E\left(Y^{\frac{k}{\delta +1}}\right)\\ & = & e^{\mu t}E\left(\sum_{k=0}^{\infty }\frac{{\left(tY\right)}^{k}}{k!}\right)\\ & = & e^{\mu t}E\left(e^{tY}\right)\\ & = & e^{\mu t}\Gamma \left(1-\sigma t\right), \end{array}$$

where Y∼GEV(0,0,σ).    □

The mean of X∼BGEV(0,μ,σ,δ) always exists. It is given in the following corollary.

**Corollary 4.**  *Let X∼BGEV(0,μ,σ,δ). The expectation of X is given by*

$$\begin{array}{c} E\left(X\right)=\mu E\left(Y^{1/(\delta +1)}\right)+{\left(-1\right)}^{\frac{\delta +2}{\delta +1}}E\left(Y^{1/(\delta +1)}I_{\left[Y<0\right]}\right)+E\left(Y^{1/(\delta +1)}I_{Y\geq 0}\right), \end{array}$$

*where Y∼GEV(0,0,σ).*

**Proof.**  The proof follows from the derivative of (23) at t=0.    □

#### 2.3.3. Entropy

The differential entropy of the BGEV distribution is given in the following proposition.

**Proposition 4.**  *Let X∼BGEV(ξ,μ,σ,δ) with ξ≥0, then the entropy of X is given by*

(28) $$\begin{array}{c} H\left(X\right)=1+\left(1+\xi \right)\gamma +\ln\left(\frac{\sigma }{\delta +1}\right)-\frac{1}{\delta +1}E\left[\ln|Y|\right], \end{array}$$

*where γ is the Euler constant and Y∼GEV(ξ,0,σ).*

**Proof.**  By definition

(29) $$\begin{array}{c} H\left(X\right)=-\int_{-\infty }^{\infty }f\left(x;\xi ,\mu ,\sigma ,\delta \right)\ln\left[f\left(x;\xi ,\mu ,\sigma ,\delta \right)\right]dx. \end{array}$$

Case ξ>0. From (9), Equation (29) becomes

(30) $$\begin{array}{ccc} H(X) & = & \int_{-\infty }^{\infty }\ln\left(\sigma \right)f\left(x;\xi ,\mu ,\sigma ,\delta \right)dx+\int_{-\infty }^{\infty }\left(\frac{1}{\xi }+1\right)\ln\left[1+\xi \frac{T(x)}{\sigma }\right]f\left(x;\xi ,\mu ,\sigma ,\delta \right)dx\\ & + & \int_{-\infty }^{\infty }{\left[1+\xi \frac{T(x)}{\sigma }\right]}^{-1/\xi }f\left(x;\xi ,\mu ,\sigma ,\delta \right)dx-\int_{-\infty }^{\infty }\ln\left(T^{\prime }\left(x\right)\right)f\left(x;\xi ,\mu ,\sigma ,\delta \right)dx. \end{array}$$

With the substitution y=T(x) in (30), it follows that

$$\begin{array}{ccc} H(X) & = & \ln\left(\frac{\sigma }{\delta +1}\right)+E{\left(1+\xi Y\right)}^{-1/\xi }+\left(\frac{1}{\xi }+1\right)E\left[\ln\left(1+\xi Y\right)\right]-\frac{1}{\delta +1}E\left[\ln|Y|\right], \end{array}$$

where Y∼GEV(ξ,0,1) is as (1). Due to the fact that $E{\left(1+\xi Y\right)}^{-1/\xi }=1$ and E[ln(1+ξY)]=ξγ, the proof of (28) is complete.

Case ξ=0. Again, from (9), the Equation (29) becomes

(31) $$\begin{array}{ccc} H(X) & = & \int_{-\infty }^{\infty }\ln\left(\sigma \right)f\left(x;0,\mu ,\sigma ,\delta \right)dx+\int_{-\infty }^{\infty }\frac{T(x)}{\sigma }f\left(x;0,\mu ,\sigma ,\delta \right)dx\\ & = & \int_{-\infty }^{\infty }e^{-\frac{T(x)}{\sigma }}f\left(x;0,\mu ,\sigma ,\delta \right)dx-\int_{-\infty }^{\infty }\ln\left(T^{\prime }\left(x\right)\right)f\left(x;0,\mu ,\sigma ,\delta \right)dx. \end{array}$$

With the substitution y=T(x), the Equation (31) is updated by

(32) $$\begin{array}{ccc} H(X) & = & \ln\left(\frac{\sigma }{\delta +1}\right)+E\left(Y\right)+E\left(e^{-Y}\right)-\frac{1}{\delta +1}E\left[\ln|Y|\right]. \end{array}$$

where Y∼GEV(0,0,1). Since E(Y)=γ and $E(e^{-Y})=1$, the Equation (32) proves (28).    □

## 3. Application

In this section, to demonstrate the applicability of the bimodal GEV model, F(·;ξ,μ,σ,δ) with PDF (9), we use data on the minimum humidity and wind gust speed of Goiânia. It is the second most populous city in the Central-West region of Brazil, surpassed only by Brasilia, the capital of Brazil. The city is an important economic hub in the region and is considered a strategic center for areas such as industry, medicine, fashion, and agriculture. In Goiânia, the climate is tropical, with a dry season with two well-defined seasons: rainy (from October to April) and dry (from May to September). In the dry season, relative humidity reaches critical levels and can be close to 10%, which constitutes a state of emergency.

The data used here correspond to the period from 1 January 2011 to 31 December 2022 and come from the automatic weather station A002 in Goiânia. Data recording is hourly. The data are available from the National Institute of Meteorology on the website https://portal.inmet.gov.br/, accessed on 15 January 2023. The relative humidity (HUM) is calculated as the percentage of water vapor in the atmosphere and the wind speed (WS) is measured in meters per second (km/h).

Table 1 shows the descriptive statistics of HUM. In the period corresponding to the data used here, the minimum humidity recorded was 21.83%.

Since the original HUM and WS data exhibit temporal dependence and the model F(·;ξ,μ,σ,δ) is for independent and identically distributed (i.i.d.) data, we first applied the minimum block technique [17] to obtain a subsample of the minimum values of HUM.

Table 2 presents a summary of the *p*-values obtained for different block sizes *N*, based on the Ljung–Box test [18] applied to the subsample of block minimum. It is observed that, from  60 towards, the *p*-values exceed the 5% significance level, indicating that for block sizes greater than or equal to 60, the null hypothesis of independence among observations is not rejected. Therefore, N=60 (1440 h) is adopted as the smallest block size that ensures serial independence within the subsample of minimum.

**Figure 5:**
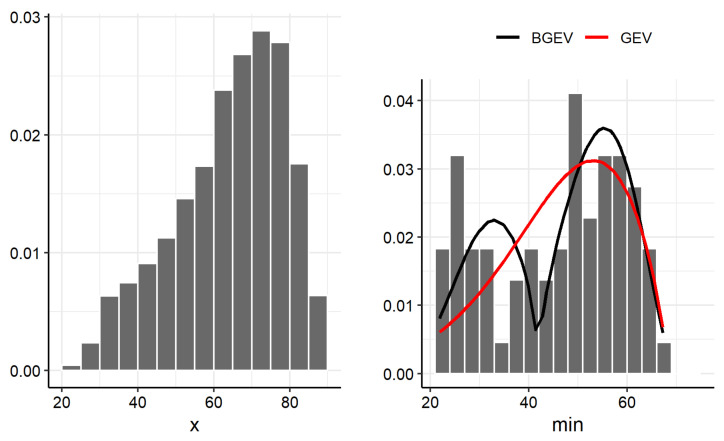
Histogram of the original HUM data (**left** panel) and histogram versus adjusted GEV and BGEV densities for HUM data (**right** panel).

**Figure 6:**
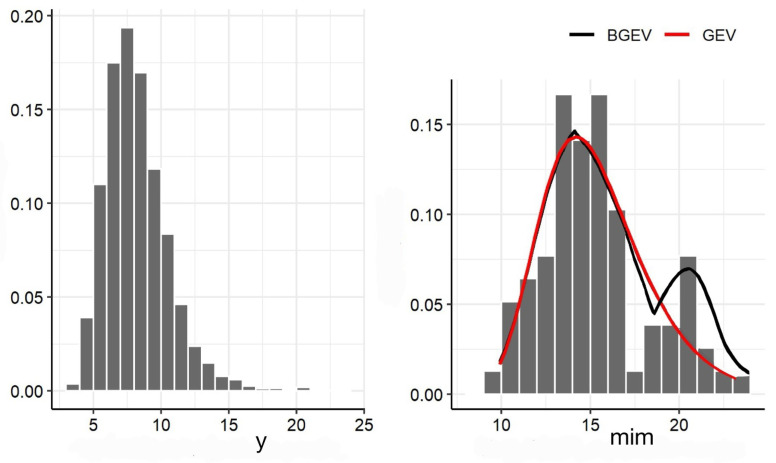
Histogram of the original WS data (**left** panel) and histogram versus adjusted GEV and BGEV densities for WS data (**right** panel).

The left panels of Figure 5 and Figure 6 display the histograms of the HUM and WS variables, respectively. The right panels of these same figures present the histograms of the subsamples composed of the minimum values of HUM and WS. The histograms of the subsamples exhibit a bimodal behavior, which suggests the use of the BGEV distribution as an appropriate model for these data. Given the extreme nature of the observations, both sets of minima were fitted using the GEV and BGEV distributions to assess the modeling ability of each distribution to capture the empirical characteristics observed.

To estimate the parameters of the GEV and bimodal GEV distributions F(·;ξ,μ,σ) and F(·;ξ,μ,σ,δ), we use the maximum likelihood technique that is implemented in the EVD [19] and bgev packages [14] in the R Project for Statistical Computing [20]. The maximum likelihood estimation algorithm of the bgev package is described in the Appendix A.

Table 3 shows the estimates and standard errors for the minimum subsamples of the HUM and WS.

The parameter δ>0 in the BGEV distribution is associated with the presence of bimodality, as previously discussed in Section 2.2. From Table 3, we observe that the estimates of δ for HUM and WS are 0.54 and 0.36, respectively. These values indicate the presence of an inherent bimodal structure in the data.

To assess the goodness of fit of the BGEV and GEV distributions to the minima of HUM and WS, we used the Akaike Information Criterion (AIC) [21]. The AIC values obtained for the HUM variable were 611.2 (BGEV) and 2550.6 (GEV), while for WS they were 834.2 (BGEV) and 3452.2 (GEV). These results indicate that the BGEV distributions provide a significantly better fit than the GEV distribution to model the minimum values of relative humidity and wind gust speed observed in Goiânia.

The Shannon entropy of a continuous random variable X∼F, denoted by H(X) and defined in Equation (29), quantifies the average uncertainty associated with the distribution *F*. In general, the higher the entropy, the greater the uncertainty of *F* in representing the observations of *X*. In the context of extreme value modeling, let $H_{1}\left(x\right)$ be the entropy of GEV(ξ,μ,σ)=BGEV(ξ,μ,σ,0) and let $H_{2}\left(x\right)$ be the entropy of BGEV(ξ,μ,σ,δ). When both the GEV and BGEV distributions are used to model extreme data, Equation (28) shows that, for δ>0, the following inequality holds:

$$H_{2}\left(x\right)<H_{1}\left(x\right),\;\;\mathrm{whenever}\;\;\frac{\sigma }{\delta +1}>1.$$

Applying this condition to the parameter estimates obtained to model the minimum HUM and WS, as presented in Table 3, we find

$$\frac{\hat{\sigma }}{\hat{\delta }+1}=41.75974\;\;\mathrm{and}\;\;\frac{\hat{\sigma }}{\hat{\delta }+1}=14.16,$$

for HUM and WS, respectively. This result implies that the entropy of the BGEV distribution is lower than that of the GEV, indicating that the BGEV model provides a more efficient fit to the data, in the sense of lower uncertainty associated with the variability of the observations.

The better performance of the BGEV distribution is further illustrated in the right panels of Figure 5 and Figure 6, which present the histograms of the adjusted GEV and BGEV densities for the minimum relative humidity and wind gust speed data, respectively.

## 4. Conclusions

The GEV distribution is a crucial tool for modeling extreme data. However, this distribution is not well suited to datasets that exhibit bimodal behavior. In this work, we examine a recent extension of the GEV distribution that accommodates bimodal data, known as the BGEV distribution, which was introduced by [13] and later redefined by [14].

In short, the main contributions of this paper are as follows. First, it presents a detailed explanation of the redefinition of the BGEV model. Second, the versatility of the BGEV distribution is illustrated through graphical representations of its PDF, which can be highly flexible, exhibiting unimodal or bimodal characteristics, as well as being symmetric or asymmetric and possessing either heavy or light tails. Third, it provides a comprehensive proof of the key properties of the new BGEV distribution, including identifiability, moments, the moment-generating function, and differential entropy. Fourth, it illustrates the usefulness of the new BGEV distribution through the application of climate data. Overall, the BGEV distribution is more effective than the GEV distribution when bimodality is inherent in the data.

A natural extension of the present work is the development of a regression model based on the BGEV distribution. The authors of this paper are currently developing a new class of regression model based on a median reparameterization of the redefined BGEV distribution discussed here. This work is in progress and the results will be reported elsewhere. Another promising extension of this work consists in developing time series models with innovations following the BGEV distribution.

A relevant limitation of the BGEV distribution arises from the fact that its support depends directly on the parameters themselves. This dependence presents challenges in optimizing the likelihood function. In the bgev package, specific strategies have been built to overcome these restrictions and ensure the numerical convergence of the algorithm, as described in Step 3 (error treatment of input parameters) of the algorithm in Appendix A. In addition, the adjustments of ξ and δ exert a strong influence on the behavior of the tails. In contexts with small sample sizes, these adjustments tend to be unstable and difficult.   

## Figures and Tables

**Table 1:** Descriptive statistics of HUM and WS.

Data	Mean	Standard Deviation	Median	Maximum	Minimum
HUM	64.00	14.54	66.56	91.12	21.83
WS	21.3	9.7	22.5	43	8.6

**Table 2:** Block size and *p*-value of Ling–Box test.

*N*	*p*-Value (HUM)	*p*-Value (WS)
55	<0.0001	<0.0001
56	0.0011	<0.0001
57	0.0010	0.0021
58	0.0023	0.0034
59	0.0039	0.0051
60	0.0059	0.0057
61	0.0050	0.0089
62	0.0071	0.0128
63	0.0101	0.0241
64	0.0134	0.0301
65	0.0222	0.0312

**Table 3:** Estimates and standard errors under the GEV and BGEV distributions for the minimum HUM and WS.

	BGEV		GEV		
**Data**	**Parameters**	**Estimates**	**Std. Error**	**Estimates**	**Std. Error**
	ξ	−0.37	0.09	−0.58	0.08
HUM	μ	41.88	0.49	43.07	1.85
	σ	64.31	2.92	14.66	1.53
	δ	0.54	0.18	-	-
	ξ	−0.06	0.14	−0.03	0.21
WS	μ	18.47	0.25	14.78	1.48
	σ	19.26	3.86	15.43	2.59
	δ	0.36	0.56	-	-

## Data Availability

The data presented in this study are available in https://bdmep.inmet.gov.br/, accessed on 10 July 2025.

## Abbreviations

    The following abbreviations are used in this manuscript:

AIC	Akaike Information Criterion
BGEV	Bimodal Generalized Extreme Value
CDF	Cumulative Distribution Function
GEV	Generalized Extreme Value
HUM	Relative Humidity
PDF	Probability Density Function

## Appendix A. MLE Procedure for BGEV Parameters

In the bgev package, the maximum likelihood estimation of the parameters ξ, μ, σ, and δ is carried out through the following steps:

### Algorithm

1. **Compute the log-likelihood:**
2. **Calculate the GEV MLE (used as initial values):**
3. **Perform BGEV MLE estimation:**

## References

[1] Fisher R.A., Tippett L.H.C. (1928). Limiting forms of the frequency distribution of the largest and smallest member of a sample. Proc. Cambridge Philos. Soc..

[2] Gnedenko B.V. (1943). Sur la distribution limite du terme maximum d’une serie aleatoire. Ann. Math..

[3] Jenkinson A.F. (1955). The frequency distribution of the annual maximum (or minimum) values of meteorological elements. Q. J. R. Meteorol. Soc..

[4] Embrechts P., Klüppelberg C., Mikosch T. (1997). Modelling Extremal Events: For Insurance and Finance.

[5] Haan L., Ferreira A. (2006). Extreme Value Theory: An Introduction.

[6] Kotz S., Nadarajah S. (2000). Extreme Value Distributions: Theory and Applications.

[7] Reiss R.-D., Thomas M. (2007). Statistical Analysis of Extreme Values with Applications to Insurance, Finance, Hydrology and Other Fields.

[8] Resnik S. (1987). Extreme Values, Regular Variation and Weak Convergence.

[9] Rudd E.M., Jain L.P., Scheirer W.J., Boult T.E. (2017). The extreme value machine. IEEE Trans Pattern Anal. Mach. Intell..

[10] Aryal G.R., Tsokos C.P. (2009). On the transmuted extreme value distribution with application. Nonlinear Anal..

[11] Nascimento F., Bourguignon M., Leão J. (2016). Extended generalized extreme value distribution with applications in environmental data. Hacettepe. J. Math. Stat..

[12] Krakauer N.Y. (2024). Extending the blended generalized extreme value distribution. Discov. Civ. Eng..

[13] Otiniano C.E.G., Paiva B.S., Vila R., Bourguignon M. (2023). A bimodal model for extremes data. Environ. Ecol. Stat..

[14] Otiniano C.E.G., Oliveira Y.L.S., Sousa T.R. (2024). Bimodal GEV Distribution with Location Parameter. https://CRAN.R-project.org/package.bgev.

[15] Gonçalves C.R., Otiniano C.E.G., Crivinel E.C. (2018). Estimation of a nonlinear discriminant function from a mixture of two GEV distributions. J. Stat. Comput. Sim..

[16] Abramowitz M., Stegun I.A. (1965). Handbook of Mathematical Functions: With Formulas, Graphs, and Mathematical Tables.

[17] Jondeau E., Poon S.H., Rockinger M. (2007). Financial Modeling Under Non-Gaussian Distributions.

[18] Box G.E.P., Pierce D.A. (1970). Distribution of residual correlations in autoregressive-integrated moving average time series models. J. Am. Stat. Assoc..

[19] Stephenson A.G. (2002). evd: Extreme value distributions. R News.

[20] R Core Team (2022). R: A Language and Environment for Statistical Computing.

[21] Akaike H. (1974). A new look at the statistical model identification. IEEE Trans. Autom. Control..

